# Glycemic dysregulation in a patient with type 2 diabetes treated with 5-azacitidine: a case report

**DOI:** 10.1186/s13256-018-1690-3

**Published:** 2018-07-03

**Authors:** Antoine Ponard, Nicole Ferreira-Maldent, Marjan Ertault, Martine Delain, Kamel Amraoui, Sandra Regina, Annie-Pierre Jonville-Béra, Olivier Hérault, Philippe Colombat, Emmanuel Gyan

**Affiliations:** 1 Service d’hématologie et thérapie cellulaire, Centre hospitalier universitaire, 2 boulevard Tonnellé, 37044 Tours, France; 2 Service de médecine interne, Centre hospitalier universitaire, Tours, France; 3 Pôle Santé Léonard de Vinci, Chambray-les-Tours, France; 4 Centre régional de pharmacovigilance, Service de pharmacologie clinique, Centre hospitalier universitaire, Tours, France; 5 Service d’hématologie biologique, Centre hospitalier universitaire, Tours, France; 6 Laboratoire LNOx, ERL CNRS 7001, Université de Tours, Tours, France; 7 Faculté de Médecine, Université François Rabelais, Tours, France

**Keywords:** Diabetes, Myelodysplastic syndrome, Hypomethylating agent, Acute myeloid leukemia

## Abstract

**Background:**

Diabetes and myelodysplastic syndrome are two conditions that may coexist in a single patient, since both diseases are prevalent in the elderly. The pathophysiology of myelodysplastic syndrome involves recurrent genetic mutations, especially in genes controlling epigenetic regulation. Although the pathophysiology of diabetes is not well understood, several studies suggest a role of epigenetics in type 2 diabetes.

**Case presentation:**

We report here for the first time the case of a 75-year-old Caucasian man who was treated for both diabetes and acute myeloid leukemia secondary to myelodysplastic syndrome, with a temporal association between glycemic dysregulation and the intake of 5-azacitidine. In fact, 2–3 days after starting each 7-day cycle of 5-azacitidine, he reported higher blood glucose levels, requiring an increased dose of self-administered insulin.

**Conclusion:**

This observation could help to understand the pathophysiology of these two conditions and could encourage physicians to monitor blood glucose levels in patients under hypomethylating agent with a history of diabetes.

## Background

The incidence of type 2 diabetes (T2D) is 5.8% in the population aged 45–64 years and reaches 13.4% in individuals over 75. The pathophysiology of T2D involves impaired insulin secretion by β-cells and insulin resistance in peripheral cells. The pathophysiology of T2D has not been fully elucidated but recent studies suggest a combination of non-genetic risk factors, such as sedentary behaviors, ageing, and obesity, and genetic risk factors [1]. There is emerging evidence for the involvement of epigenetic regulation in the pathophysiology of diabetes [1, 2]. Epigenetic regulation is the control of gene expression without changes in desoxynucleic acid (DNA) sequence, through the modification of DNA methylation and histone acetylation. DNA methylation, catalyzed by DNA methyltransferases (DNMTs), occurs on the cytosines of CpG dinucleotides, resulting in the generation of 5-methylcytosine. CpG hypermethylation of promoter regions results in low chromatin access to transcription factors and gene expression inhibition. Cancer cells exhibit distinct regions of DNA hypermethylation in promoters of tumor-suppressor genes.

Myelodysplastic syndromes (MDS) are clonal myeloid malignancies characterized by ineffective hematopoiesis resulting in blood cytopenia. MDS are more common in the elderly, with a median age at diagnosis ranging between 65 and 70 years and an incidence of 1.75/100,000 and 37.8/100,000 in individuals aged 50–54 and 75–79 years, respectively. In MDS, myeloid progenitor cells carry one or more recurrent point mutations in genes involved in epigenetic regulation, leading to transcription silencing of genes [3]. *DNMT3A*, isocitrate deshydrogenase-1 and 2 or TET-2 methylcytosine dioxygenase genes are often mutated in patients with high-risk MDS, and are involved in skewing DNA methylation patterns.

5-azacitidine is a structural analogue of cytosine that incorporates into DNA and covalently inhibits DNMT. 5-azacitidine has been approved for the treatment of patients with higher-risk MDS and acute myeloid leukemia (AML) based on the results of a phase 3 study showing a survival advantage compared to conventional treatment [4]. We describe a case of glycemic dysregulation in a patient with diabetes treated with 5-azacitidine for AML.

## Case presentation

**Fig. 1:**
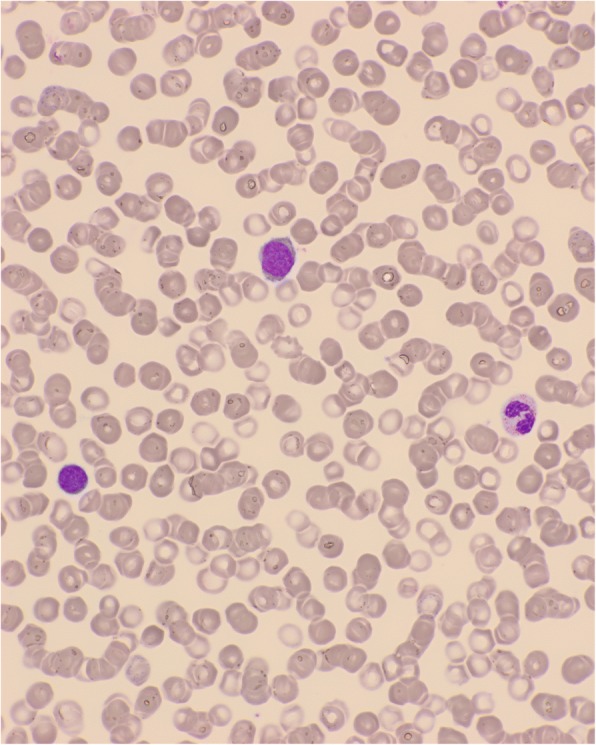
Blood smear at diagnosis showed low blast percentage and dyserythropoiesis

**Fig. 2:**
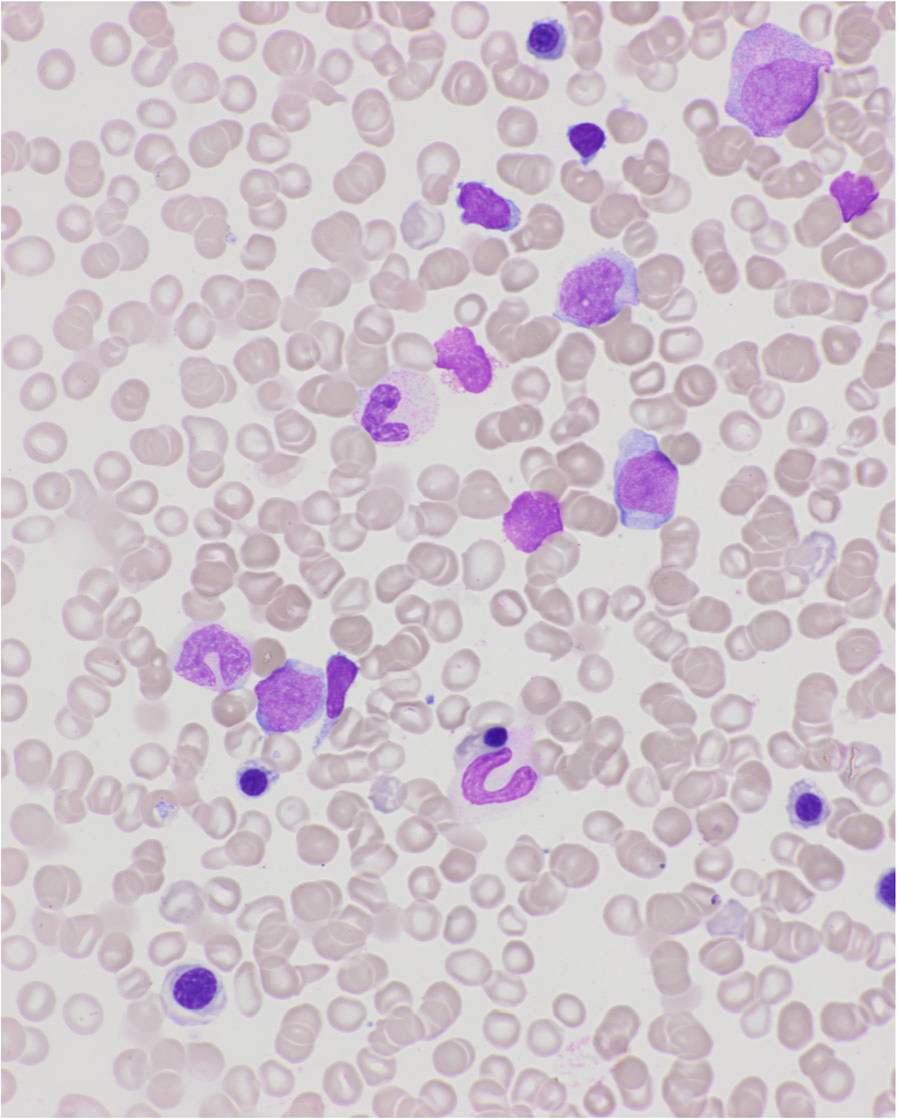
Marrow smear at diagnosis, showing low blast percentage

A 75-year-old Caucasian man was admitted for bicytopenia in May 2013. His blood cell count was as follows: hemoglobin 8 g/dl, platelets 87 × 10^9^/l, and leukocytes 6.1 × 10^9^/L (Fig. 1). His medical history included T2D treated with biphasic insulin aspart, 50 units at breakfast and 30 units at bedtime, and the dose of rapid-acting insulin was adjusted according to his blood glucose level at lunch. He was also followed for triple-bypass surgery for coronary disease in 2010. No family history of hematological malignancies was noted. Initially, he was hospitalized in an intensive care unit for grade IV anemia at 5.5 g/dl. After blood transfusions, he was admitted in our hematology department. Bone marrow aspiration showed multilineage dysplasia with 8% of blasts (Fig. 2), consistent with a diagnosis of MDS with excess blasts: refractory anemia with excess blasts-1 (RAEB-1). A karyotype analysis identified trisomy 13. Immunophenotypic evaluation showed the presence of CD34+, CD117+, cytoplasmic myeloperoxidase (CMPO+), and CD33− cells. Plasma folate, vitamin B12, iron, and thyroid stimulating hormone levels were normal. No inflammatory syndrome was found. He was initially treated with darbepoetin 300 μg per week, without response.

**Fig. 3:**
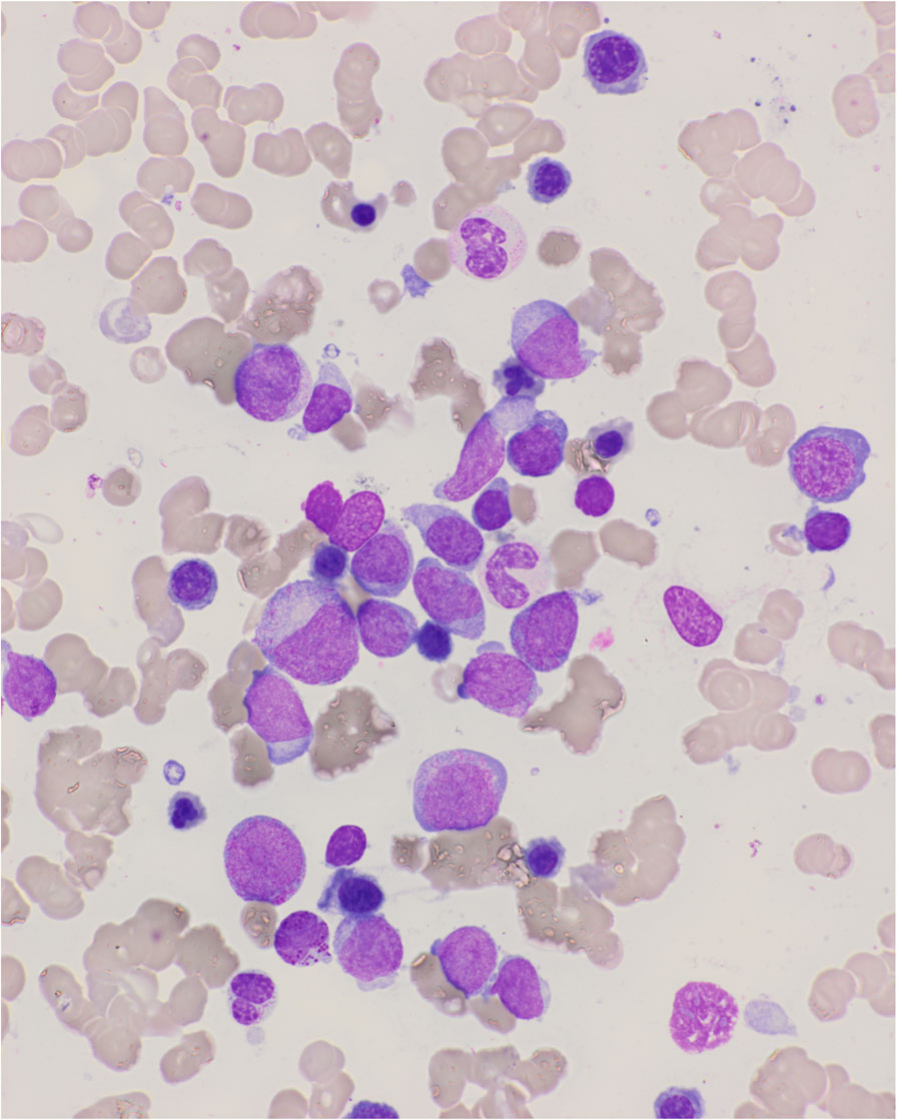
Marrow smear at progression showing high blast percentage and dyserythropoiesis

**Fig. 4:**
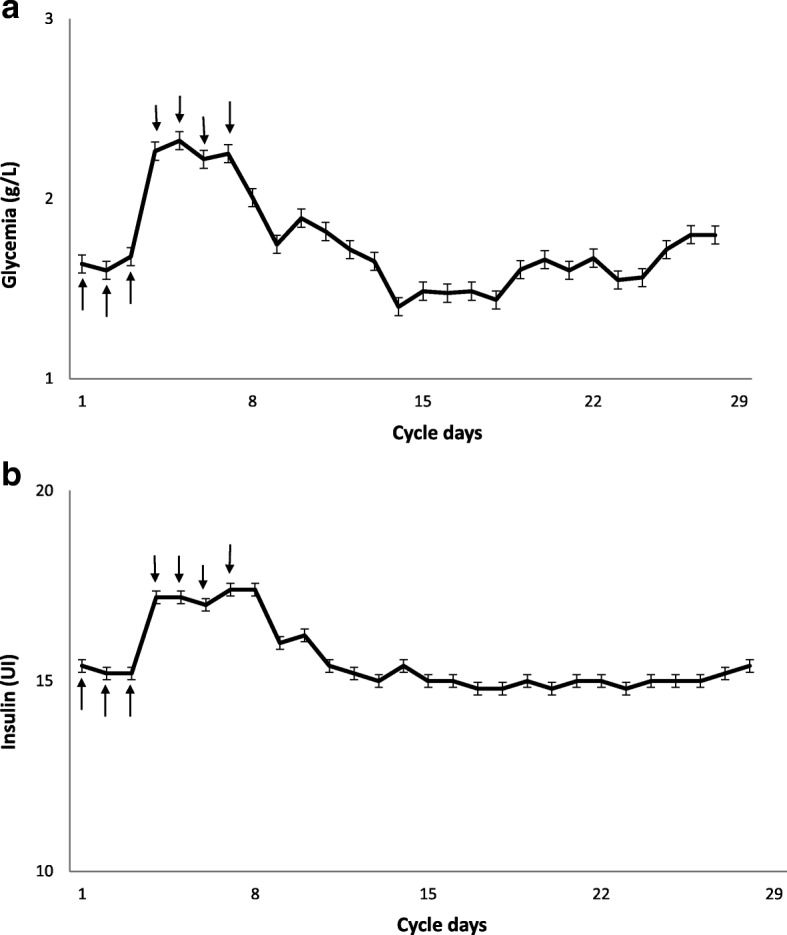
**a** Daily mean fasting blood glucose levels from cycles 14 to 19 of 5-azacitidine were recorded. The *continuous line* represents the mean values for each cycle. The *error bars* indicate the standard deviation of measurements. **b** Morning rapid-acting insulin doses. The continuous line represents the mean dose of rapid-acting insulin administered from cycles 14 to 19. The *arrows* indicate the days of 5-azacitidine injection days 1 to 7

Two months later, because of a worsening of cytopenia, a second bone marrow aspiration was performed. It showed 29% of blasts, consistent with the diagnosis of AML progression (Fig. 3). He consented to participate in a phase I–II clinical research study assessing the combination of 5-azacitidine with idarubicin in July 2013. He showed a partial response with 8% of bone marrow blasts after six cycles, and transfusion independence. After four cycles of 5-azacitidine, he reported higher blood glucose levels 2–3 days after the start of each 5-azacitidine cycle, requiring an increased dose of self-administered insulin. No change in diet or in physical activities or other causes of glycemic dysregulation such as corticosteroids were noted. Further monitoring confirmed higher blood glucose levels from days 4 to 12 of each 5-azacitidine cycle (Fig. 4). Our patient experienced such dysregulation during all the additional 15 cycles until a fatal progression of AML.

## Discussion

To the best of our knowledge, this is the first case of this potential side effect of 5-azacitidine reported so far. Such a metabolic effect has been reported with decitabine [5], another hypomethylating agent. Several cases of hyperglycemia have been reported in a clinical trial assessing 5-azacitidine [6]. However, the systematic temporal association seen in our case has not been reported to date. Thus, we assumed that 5-azacitidine could reversibly impair glucose regulation mechanisms. The mechanism of such dysregulation has not been fully identified. In our opinion, 5-azacitidine could impair β-cell function in pancreatic islets through an epigenetic mechanism. Dayeh *et al.* have analyzed the genome-wide DNA methylation pattern in pancreatic islets isolated from patients with T2D as well as non-diabetic patients [2]. The methylome analysis identified 1649 CpG sites with significantly different DNA methylation levels. Decreased methylation was observed in 1596 of these CpG sites and was associated with increased gene expression in T2D patient islets. Overexpression of some hypomethylated genes was associated with a significant decrease in glucose-induced insulin secretion by β-cells [2]. We hypothesized that 5-azacitidine injections induced hypomethylation in pancreatic islets and led to overexpression of such genes.

## Conclusions

In the case described here, the temporal association between higher blood glucose levels and the initiation of 5-azacitidine, with an improvement after each cycle is consistent with a causative role of this drug. We assumed that the epigenetic pathway could be the link between 5-azacitidine and the glycemic dysregulation. This observation could encourage physicians to carefully monitor blood glucose levels in patients under hypomethylating agents with a history of diabetes.
